# Hepatic B cell leukemia-3 suppresses chemically-induced hepatocarcinogenesis in mice through altered MAPK and NF-κB activation

**DOI:** 10.18632/oncotarget.10893

**Published:** 2016-07-28

**Authors:** Nadine Gehrke, Marcus A. Wörns, Amrit Mann, Yvonne Huber, Nadine Hoevelmeyer, Thomas Longerich, Ari Waisman, Peter R. Galle, Jörn M. Schattenberg

**Affiliations:** ^1^ Department of Medicine and University Medical Center of the Johannes Gutenberg University, Mainz, Germany; ^2^ Institute for Molecular Medicine, University Medical Center of the Johannes Gutenberg University, Mainz, Germany; ^3^ Institute of Pathology, University Hospital RWTH Aachen, Aachen, Germany

**Keywords:** B cell leukemia-3 (Bcl-3), hepatocellular carcinoma (HCC), mitogen-activated protein kinase (MAPK), apoptosis, nuclear factor kappa B (NF-kB)

## Abstract

The transcriptional nuclear factor kappa B (NF-κB)-coactivator B cell leukemia-3 (Bcl-3) is a molecular regulator of cell death and proliferation. Bcl-3 has been shown to be widely expressed in different cancer types including hepatocellular carcinoma (HCC). Its influence on hepatocarcinogenesis is still undetermined. To examine the role of Bcl-3 in hepatocarcinogenesis mice with hepatocyte-specific overexpression of Bcl-3 (Bcl-3^Hep^) were exposed to diethylnitrosamine (DEN) and phenobarbital (PB). Hepatic Bcl-3 overexpression attenuated DEN/PB-induced hepatocarcinogenesis. Bcl-3^Hep^ mice exhibited a lower number and smaller tumor nodules in response to DEN/PB at 40 weeks of age. Reduced HCC formation was accompanied by a lower rate of cell proliferation and a distinct expression pattern of growth and differentiation-related genes. Activation of c-Jun N-terminal kinase (JNK) and especially extracellular-signal regulated kinase (ERK) was reduced in tumor and tumor-surrounding liver tissue of Bcl-3^Hep^ mice, while p38 and NF-κB p65 were phosphorylated to a higher extent compared to the wild type. In parallel, the absolute number of intrahepatic macrophages, CD8^+^ T cells and activated B cells was reduced in DEN/PB-treated Bcl-3^Hep^ mice mirroring a reduction of tumor-associated inflammation. Interestingly, at the early time point of 7 weeks following tumor initiation, a higher rate of apoptotic cell death was observed in Bcl-3^Hep^ mice. In summary, hepatocyte-restricted Bcl-3 overexpression reduced hepatocarcinogenesis related to prolonged liver injury early after tumor initiation likely due to decreased survival of DEN/PB-damaged, premalignant cells. Therefore, Bcl-3 could become a novel player in the development of therapeutic and diagnostic tools for HCC.

## INTRODUCTION

Hepatocellular carcinoma (HCC) is the fifth most frequent cancer worldwide and ranks third in overall cancer mortality. Its incidence has continued to rise in recent years in western countries due to infection with hepatitis B and C, alcohol-induced liver diseases, but also non-alcoholic steatohepatitis (NASH) [1]. Curative treatment can rarely be achieved and additional therapeutic options are urgently required. Therefore, the mechanisms underlying the initiation and progression of HCC have to be better understood in order to develop novel and drugable molecular targets.

Accumulating evidence indicates that chronic inflammation leading to hepatocellular injury is an initiating factor during HCC formation. Hepatic inflammation results from the continued secretion of cytokines by dying hepatocytes activated hepatic stellate cells, and Kupffer cells that lead to the recruitment of inflammatory cells. This is a crucial step that triggers hepatic regeneration and proliferation of progenitor cells to restore the hepatic tissue. The sustained cycle of necroinflammation and hepatocyte regeneration is thought to provide the mitogenic and mutagenic microenvironment that promotes the formation of dysplastic nodules and HCC [2]. On the opposite, increased apoptosis of transformed cells reduces tumor burden and tumor directed cell death was shown to be an effective treatment option for HCC. Currently the only licensed therapy for advanced HCC is the multi-tyrosine kinase inhibitor Sorafenib that promotes inflammation and cell death in malignant and transformed hepatocytes.

Recent evidence implicates a pivotal role for nuclear factor kappa B (NF-κB) in hepatocarcinogenesis. This nuclear transcription factor is a key regulator of inflammation and cell proliferation in the liver and belongs to a family of proteins including p65 (RelA), c-Rel (Rel), RelB, p50/p105 (NF-κB1) and p52/p100 (NF-κB2). These proteins form homo- or heterodimers complexes that are retained in the cytoplasm through binding of inhibitory IκB proteins including among others B cell leukemia-3 (Bcl-3) [3, 4]. Following degradation of the inhibitory IκB proteins, NF-κB dimers translocate from the cytoplasm to the nucleus, binding to κB enhancer elements of target genes and initiating the transcription of genes controlling inflammation, immunity, wound healing, acute phase responses, proliferation and apoptosis [5]. The loss of NF-κB promotes hepatocarcinogenesis involving increased inflammation and cell turn-over, however environmental and cell type-specific differences can influence NF-κB function. [2]. Likewise, loss of the regulatory subunits, e.g. IKKγ/NEMO in hepatocytes, was shown to lead to severe necroinflammation and HCC formation from sustained NF-κB activation [6]. Interestingly, the timing of NF-κB and IKK inhibition appears to be crucial for the physiological effect. In the Mdr-2 knockout-model of biliary inflammation, inactivation of NF-κB at a late but not early stage mitigated tumor formation [7].

Bcl-3, which was originally identified in a subset of B cell chronic lymphocytic leukemia [8], is highly expressed in the liver [9]. It binds tightly to the NF-κB subunits p50 and p52 homodimers and both activation and inhibition of the transcription of NF-κB-dependent genes have been described [9–11]. However, some functions of Bcl-3 seem to be mediated through interaction with non-NF-κB related proteins including the retinoic acid receptor or AP1 [12]. Bcl-3 expression has been described in a variety of cancer at different stages, including breast [13], nasopharyngeal [14] and pancreatic cancer [15] as well as lymphoma [16] and HCC [17]. In HCC, overexpression of Bcl-3 and increased nuclear expression of the NF-κB subunits p50 and p52 has been described [18]. *In vitro*, Bcl-3-p52 heterodimers promote the transcription of genes encoding the cell cycle regulator cyclin D1 and the anti-apoptotic Bcl-2 protein, suggesting one potential oncogenic mechanism [19, 20]. Recently, a protective role of Bcl-3 was observed in the inflammatory DSS-model of colon cancer [21]. Overall, the tumorigenic potential of NF-κB regulating cofactors appears to be cell type-specific and thus exploration in well-defined models is required.

To explore the mechanistic role of Bcl-3 during hepatocarcinogenesis, we used the two-stage diethylnitrosamine (DEN)/phenobarbital (PB) model of liver cancer in transgenic mice exhibiting a hepatocyte-specific overexpression of Bcl-3 (Bcl-3^Hep^) and corresponding littermate wild type controls. Overexpression of hepatic Bcl-3 attenuated DEN/PB-induced hepatocarcinogenesis. Reduced tumor formation in Bcl-3^Hep^ mice at 40 weeks of age was associated with a decrease in cell proliferation and inflammation in the liver, and accompanied by reduced injurious mitogen-activated protein kinases (MAPK) and enhanced NF-κB signaling pathways. Bcl-3 expression in hepatocytes lowered the resistance against DEN/PB-induced apoptotic cell death, increasing the rate of cellular injury during the early phase of HCC initiation and through this potentially diminished the number of transformed cells. Thus, these data point towards an important protective role of hepatocellular Bcl-3 during inflammatory hepatocarcinogenesis.

## RESULTS

### Hepatocyte-specific overexpression of Bcl-3 protects from DEN/PB-induced HCC development

**Figure 1:**
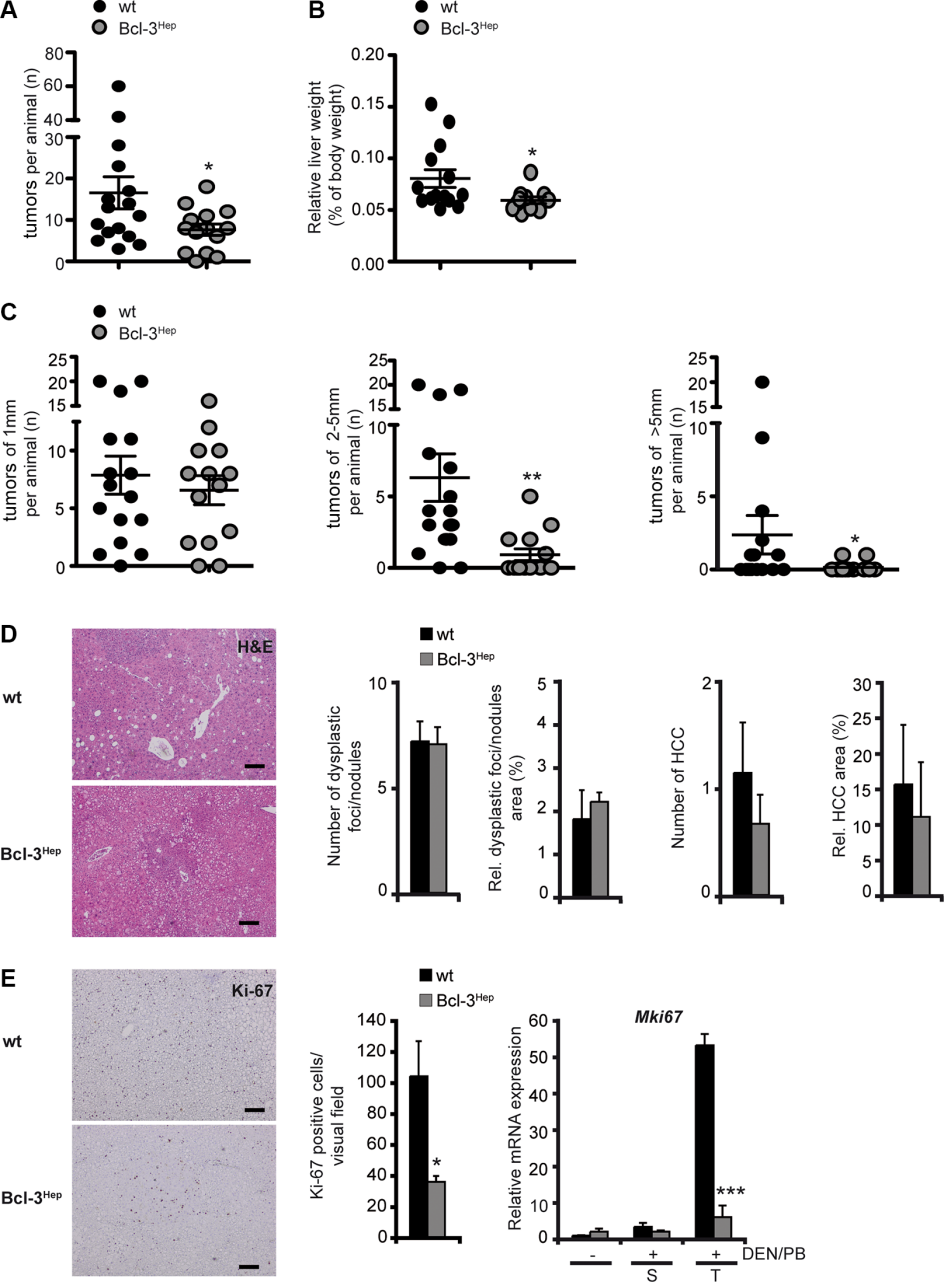
Overexpression of hepatic Bcl-3 attenuated DEN/PB-induced HCC in mice Hepatocarcinogenesis was induced by a single i.p. injection of diethylnitrosamine (DEN) in male Bcl-3Hep mice and wild type (wt) littermates 7–10 days post-partum followed by continuous treatment with phenobarbital (PB). Livers were analyzed at 40 weeks of age. (**A**) Nodules visible at the surface upon macroscopic examination, (**B**) relative liver weight and (**C**) diameter of the visible nodules. (**D**) Standard H&E stainings (scale bar: 2000 μm, representative histological stains) were scored in a blinded fashion. (**E**) Ki-67 stainings (scale bar: 2000 μm, representative histological stains), quantification and relative mRNA expression analyses in tumor (T) and surrounding (S) liver tissue of DEN/PB-treated mice and in the liver of untreated controls. Data in A–E are means of *n* = 7–16 mice/group ± SEM. *p* values for wt vs. Bcl-3Hep mice: *p < .05, **p < .01, ***p < .001.

To investigate the role of Bcl-3 in hepatocarcinogenesis, 7–10 day-old, male Bcl-3^Hep^ mice exhibiting Bcl-3-specific overexpression in hepatocytes and wild type littermates received a single i.p. dose of DEN followed by continuous treatment with PB. Tumor formation was assessed at 40 weeks of age. Except for one Bcl-3^Hep^ mouse, all mice developed macroscopically detectable tumor nodules on the liver surface. In total Bcl-3^Hep^ mice exhibited a significantly reduced number of tumors compared to wild type mice in response to DEN/PB (Bcl-3^Hep^ vs. wt: 7.64 ± 1.39 vs. 16.56 ± 3.86, *p* < 0.05, Figure 1A). The average number of tumor nodules per animal was approximately 2-fold lower and the relative liver weight of Bcl-3^Hep^ mice was significantly lower compared to the wild type (Figure 1B, Table 1). The most striking feature was that Bcl-3^Hep^ mice had dramatically smaller tumors compared to the wild type. The average size of tumor nodules in wild type mice was 2.9 mm, while the average size in Bcl-3^Hep^ mice was only 1.4 mm (*p* < 0.01, Figure 1C). Histopathological tumor grading by microscopic analyses of hematoxylin and eosin (H&E) stained liver sections revealed a comparable number of dysplastic foci and nodules in all liver sections irrespective of the genotype (Figure 1D). However, there was an increased frequency of HCC and an increased relative area of HCC in wild type compared to Bcl-3^Hep^ mice. Furthermore, cell proliferation as assessed by Ki-67 staining and qRT-PCR analyses of *Mki67* encoding Ki-67 was significantly reduced in tumorous lesions of Bcl-3^Hep^ compared to wild type mice (Figure 1E). Thus, overexpression of Bcl-3 in hepatocytes attenuated DEN/PB-induced HCC formation.

**Table 1:** Body weight, relative liver weight, liver serum parameters and caspase 3 activity in the different experimental groups at 40 weeks of age

	wt untreated	Bcl-3^Hep^ untreated	wt + DEN/PB	Bcl-3^Hep^ + DEN/PB
**Body weight (g)**	39.38 ± 1.10	33.71 ± 2.47	39.36 ± 1.78	33.49 ± 2.05 (*)
**Relative liver weight**	0.039 ± 0.000	0.032 ± 0.001	0.081 ± 0.008	0.059 ± 0.003 (*,$$)
**ALT (U/l)**	24.33 ± 2.42	41.00 ± 3.77 (*)	64.38 ± 18.19	55.60 ± 14.59
**AST (U/l)**	116.33 ± 14.23	175.88 ± 11.09 (*)	160.62 ± 18.32	159.50 ± 15.84
**LDH (U/l)**	252.00 ± 3.62	202.67 ± 0.57	823.67 ± 117.95	388.00 ± 55.36 (**,$)
**Caspase 3 activity**	111.55 ± 6.42	108.33 ± 3.88	185.26 ± 14.1	217.07 ± 10.87

Data are expressed as mean of *n* = 7–16 mice/group ± SEM. *p* values for wt vs. Bcl-3^Hep^ mice: *p < .05, **p < .01, and wt/Bcl-3^Hep^ mice untreated (− DEN/PB) vs. treated (+ DEN/PB): ^$^*p* < .05, ^$$^*p* < .01.

### Bcl-3 overexpression promotes liver injury early following tumor initiation

**Figure 2:**
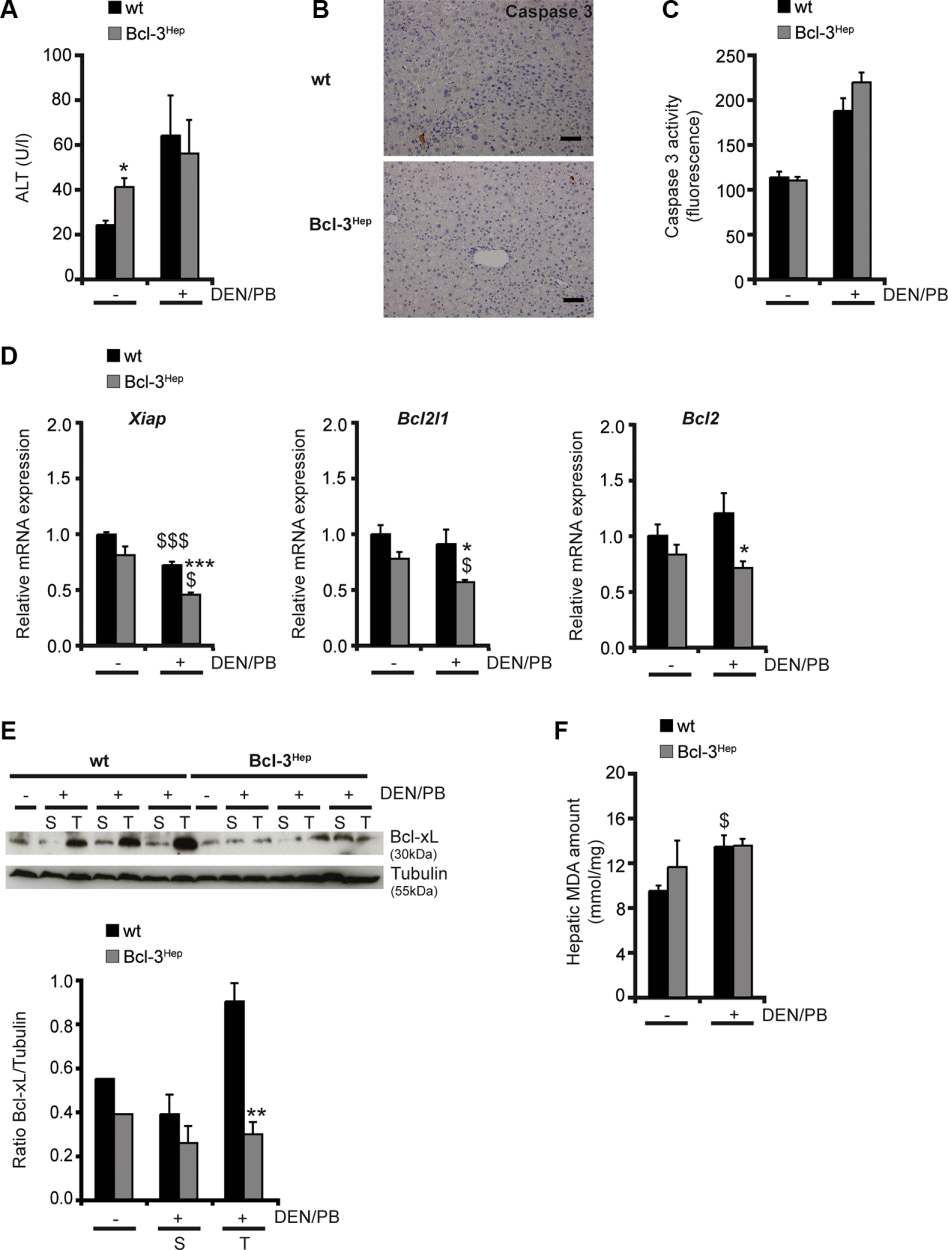
Liver injury following DEN/PB-treatment (**A**) Serum ALT levels, (**B**) immunohistochemistry for activated (cleaved) caspase 3 (scale bar: 2000 μM, representative histological stains), (**C**) caspase 3 activity measurement, (**D**) relative hepatic Xiap, Bcl2l1 and Bcl2 mRNA expression, (**E**) hepatic Bcl-xL protein expression and (**F**) amount of malondialdehyde (MDA) in the liver of 40-weeks-old DEN/PB-treated Bcl-3^Hep^ and wt mice and untreated controls. Data in A, C and E are means of *n* = 7–16 mice/group ± SEM. *p* values for wt vs. Bcl-3^Hep^ mice: *p < .05, **p < .01, ***p < .001 and wt/Bcl-3^Hep^ mice untreated (− DEN/PB) vs. + DEN/PB: ^$^*p* < .05, ^$$$^*p* < .001. In E representative immunoblots of untreated (−) and treated (+) mice with samples derived from tumor (T) and surrounding (S) tissue are shown. Tubulin served as protein loading control.

To explore the mechanisms underlying protection form hepatocarcinogenesis, Bcl-3 overexpressing mice were examined at different time points. Previous studies have shown that Bcl-3 regulates genes involved in hepatic lipid metabolism and aggravates the development steatohepatitis on a high fat diet [22]. mRNA Seq and subsequent KEGG-pathway analyses, revealed that *Mgll* (monoacylglycerol lipase, *p* = 5.51e^−11^) – a gene that inhibits cellular proliferation and reduces hepatic and colorectal cancer growth [23] – was significantly up-regulated in Bcl-3^Hep^ mice [22]. Up until the age of 72 weeks no spontaneous tumor formation was observed (data not shown), despite elevated transaminases at the age of 40 weeks (Figure 2A, Table 1), a dysmetabolic phenotype with impaired glucose tolerance and mild steatohepatitis [22]. DEN/PB-induced cell death at that time point seemed to be caspase-mediated. Immunohistochemistry and enzymatic assay showed marginal elevated, but comparable activity of caspase 3 in both genotypes (Figure 2B and 2C, Table 1). Following DEN/PB-exposure Bcl-3^Hep^ mice showed a significantly reduced mRNA expression of anti-apoptotic factors including *Xiap* (*p* < 0.001), *Bcl2l1* (*p* < 0.05) and *Bcl2* (*p* < 0.05) in the liver (Figure 2D) in parallel to increased rates of apoptosis in tumor tissue. In contrast, tumorous lesions in wild type mice exhibited enhanced levels of anti-apoptotic Bcl-xL protein (Figure 2E), supporting an unfavorable tumor phenotype. These data indicate that Bcl-3 inhibits pro-survival factors and promotes cellular injury during DEN/PB-induced hepatocarcinogenesis.

To assess the early phase of tumor initiation, DEN/PB-exposed mice were examined at the age of 4, 5 and 7 weeks. In week 4 and 5 following DEN/PB-treatment transaminases were elevated comparably in both genotypes (Bcl-3^Hep^ vs. wt: 60.0 ± 12.7 vs. 67.7 ± 10.6 U/l and 53.0 ± 3.0 vs. 51.2 ± 11.2 U/l, *n* = 6 mice/group). At week 7 ALT decreased to normal levels in the wild type while Bcl-3^Hep^ mice showed persistently elevated ALT levels indicative of prolonged liver injury (Bcl-3^Hep^ vs. wt: 50.4 ± 1.9 vs. 20.0 ± 1.3 U/l, *p* < 0.001, *n* = 6 mice/group, Table 2). In parallel, DEN/PB-treated Bcl-3^Hep^ mice displayed increased caspase 3 activity accompanied by significantly higher levels of *Tnf* and *Ccl2* mRNA compared to the wild type (Table 2). Importantly, levels of *Bcl3* at this age were elevated two-fold (data not shown).

**Table 2:** Body weight, relative liver weight, liver serum parameters, caspase 3 activity and relative hepatic TNF and CCL2 expression in the different experimental groups at 7 weeks of age

	wt untreated	Bcl-3^Hep^ untreated	wt + DEN/PB	Bcl-3^Hep^ + DEN/PB
**Body weight (g)**	22.53 ± 1.57	18.33 ± 1.12	23.50 ± 0.51	17.46 ± 0.42 (***)
**Relative liver weight**	0.063 ± 0.004	0.061 ± 0.004	0.058 ± 0.001	0.058 ± 0.002
**ALT (U/l)**	16.67 ± 2.72	19.33 ± 0.54	20.00 ± 1.33	50.40 ± 1.91 (***)
**AST (U/l)**	114.00 ± 38.72	122.67 ± 10.89	139.00 ± 13.39	144.80 ± 25.50
**LDH (U/l)**	342.00 ± 116.17	358.00 ± 31.84	810.00 ± 196.16	826.40 ± 251.36
**Caspase 3 activity**	73.05 ± 1.66	81.65 ± 6.26	81.78 ± 8.54	104.15 ± 1.33 ($)
**Hepatic TNF mRNA expression**	1.00 ± 0.03	0.69 ± 0.01	0.64 ± 0.06	0.88 ± 0.12 (*)
**Hepatic CCL2 mRNA expression**	1.00 ± 0.01	0.92 ± 0.03	0.85 ±0.10	1.45 ± 0.13 (*)

Data are displayed as mean of *n* = 3–6 mice/group ± SEM. *p* values for wt vs. Bcl-3Hep mice: *p < .05, ***p < .001 and wt/Bcl-3^Hep^ mice untreated (− DEN/PB) vs. treated (+ DEN/PB): ^$^*p* < .05.

The mechanism of DEN/PB-induced hepatocarcinogenesis involves DNA damage and hepatocellular injury from excessive oxidative stress. However, no differences in malondialdehyde (MDA) levels - an end product of lipid peroxidation - were detectable in both genotypes at 40 weeks of age (Figure 2E). Together, these data suggest that hepatocyte-specific overexpression of Bcl-3 protects from HCC-formation through prolonged cellular injury and reduced survival of transformed cells in Bcl-3 overexpressing mice.

### p38 MAPK and NF-κB pathways antagonize JNK- and ERK-signaling in DEN/PB-treated Bcl-3^Hep^ mice

**Figure 3:**
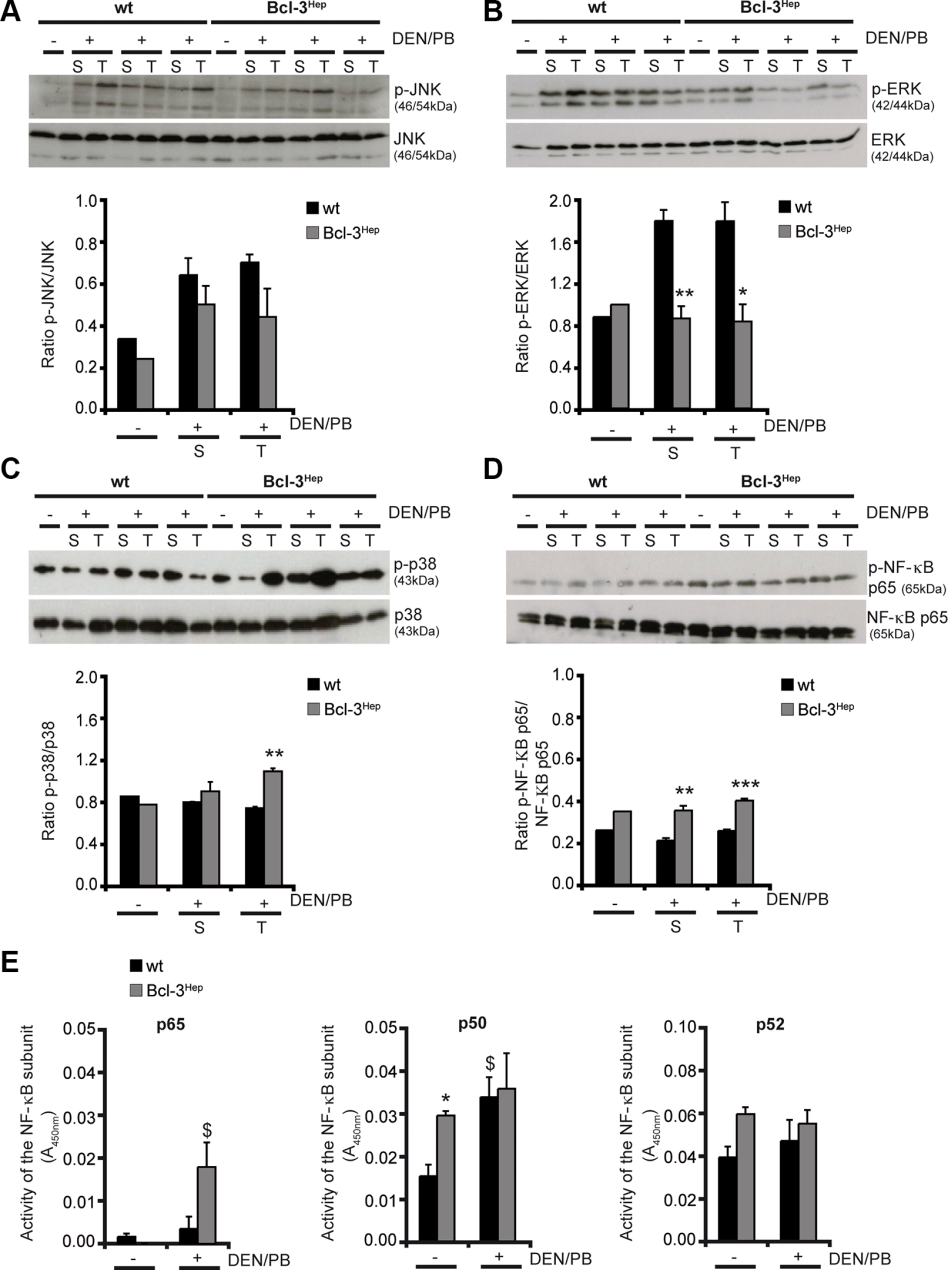
Altered MAPK- and NF-κB-signaling in DEN/PB-treated Bcl-3^Hep^ mice (**A**) Activation of JNK, (**B**) ERK, (**C**) p38 and (**D**) NF-κB p65 was determined by immunoblotting of phosphorylated JNK/ERK/p38/NF-κB p65 and total JNK/ERK/p38/NF-κB p65 protein in tumor (T) and surrounding (S) liver tissue of 40-weeks-old DEN/PB-treated (+) Bcl-3^Hep^ and wt mice and in the liver of untreated (−) controls. (**E**) Activity of the NF-κB subunits p65, p50 and p52 in the liver of DEN/PB-treated mice and untreated controls. In A–D representative immunoblots are shown. Data in E are means of *n* = 7–16 mice/group ± SEM. *p* values for wt vs. Bcl-3^Hep^ mice: *p < .05, **p < .01, ***p < .001 and wt/Bcl-3^Hep^ mice untreated (− DEN/PB) vs. treated (+ DEN/PB): ^$^*p* < .05.

Stress-activated mitogen-activated protein kinase (MAPK) signaling conveying on c-Jun N-terminal kinase (JNK) has been implicated in the growth of carcinogen- and ROS-induced HCC by promoting an inflammatory hepatic environment that supports hepatocyte proliferation and tumor development [24]. Consistent with this, mice exhibited an increased phosphorylation of the p46 and p54 isoforms of JNK in tumor and surrounding liver tissue in response to DEN/PB (Figure 3A). JNK activation was slightly more pronounced in tumorous lesions of wild type compared to Bcl-3^Hep^ mice. Also activation of the MAPK extracellular-signal regulated kinase (ERK), which plays a central role in cellular growth and differentiation [25], was significantly more pronounced in tumor (*p* < 0.05) and surrounding liver tissue (*p* < 0.01) of wild type compared to Bcl-3^Hep^ mice (Figure 3B). In contrast, the MAPK p38, which can be activated following inhibition of ERK activity [26] and negatively regulates cell cycle progression at the G1/S and the G2/M transitions by antagonizing the JNK-pathway, down-regulation of cyclins, up-regulation of cyclin-dependent kinase (CDK) inhibitors, and modulation of p53 [27], was phosphorylated and activated to a higher degree in tumor tissue of Bcl-3^Hep^ mice (*p* < 0.01) compared to the wild type (Figure 3C).

Previously, Bcl-3 was shown to act as a coactivator of NF-κB, which also prevents prolonged JNK activation and hepatocyte death [28]. Consistently, the NF-κB subunit p65 was significantly more phosphorylated in the liver of Bcl-3^Hep^ mice compared to the wild type (*p* < 0.001 in tumor and *p* < 0.01 in surrounding liver tissue) (Figure 3D) and p65 activity was significantly elevated in Bcl-3^Hep^ livers compared to wild type livers due to DEN/PB (Figure 3E). Both NF-κB p50 and p52, which are regulated by Bcl-3, participate in target gene transactivation and form heterodimers with p65. Bcl-3^Hep^ mice exhibited a markedly higher activity of p50 and p52 compared to the wild type (Figure 3E). In response to DEN/PB p50 activity increased in both genotypes, whereas p52 activity was unaffected. These data suggest that the mechanisms by which Bcl-3^Hep^ mice are less susceptible to DEN/PB-induced hepatocarcinogenesis involves inhibition of the MAPK ERK and activation of both p38 and NF-κB p65, which act to block prolonged JNK activation.

To replicate these data *in vitro*, hepatocytes from wild type and Bcl-3^Hep^ mice were isolated and treated *ex vivo* with DEN. Remarkably, decreased cellular viability was observed only in DEN-treated hepatocytes derived from Bcl-3^Hep^ mice but not wild type hepatocytes (Supplementary Figure 1A). This occurred in parallel to JNK and ERK phosphorylation, which was more pronounced in wild type hepatocytes (Supplementary Figure 1B and 1C). Additionally, DEN-treated Bcl-3^Hep^ hepatocytes exhibited a higher activation of p38 (Supplementary Figure 1D), while levels of NF-kB p65 were unaffected (Supplementary Figure 1E), comparable to the *in vivo* findings (Supplementary Figure 2A–2C). Interestingly, at week 7 p38 activity was unaffected. To assess the functional role of MAPK activation, pretreatment with the pan caspase inhibitor zVAD, the JNK-inhibitor SP600125 or the p38-inhibitor SB203580 were performed. Inhibitor pretreatment abrogated cell death in Bcl-3^Hep^ hepatocytes, suggesting that DEN-induced hepatocellular apoptosis is caspase-dependent and involves JNK- and p38-signaling, while the NF-κB inhibitor BAY11-7082 did not rescue Bcl-3^Hep^ hepatocytes (Supplementary Figure 1A).

### Hepatic Bcl-3 regulates proliferation and cell cycle progression and suppresses oncogenic signals

**Figure 4:**
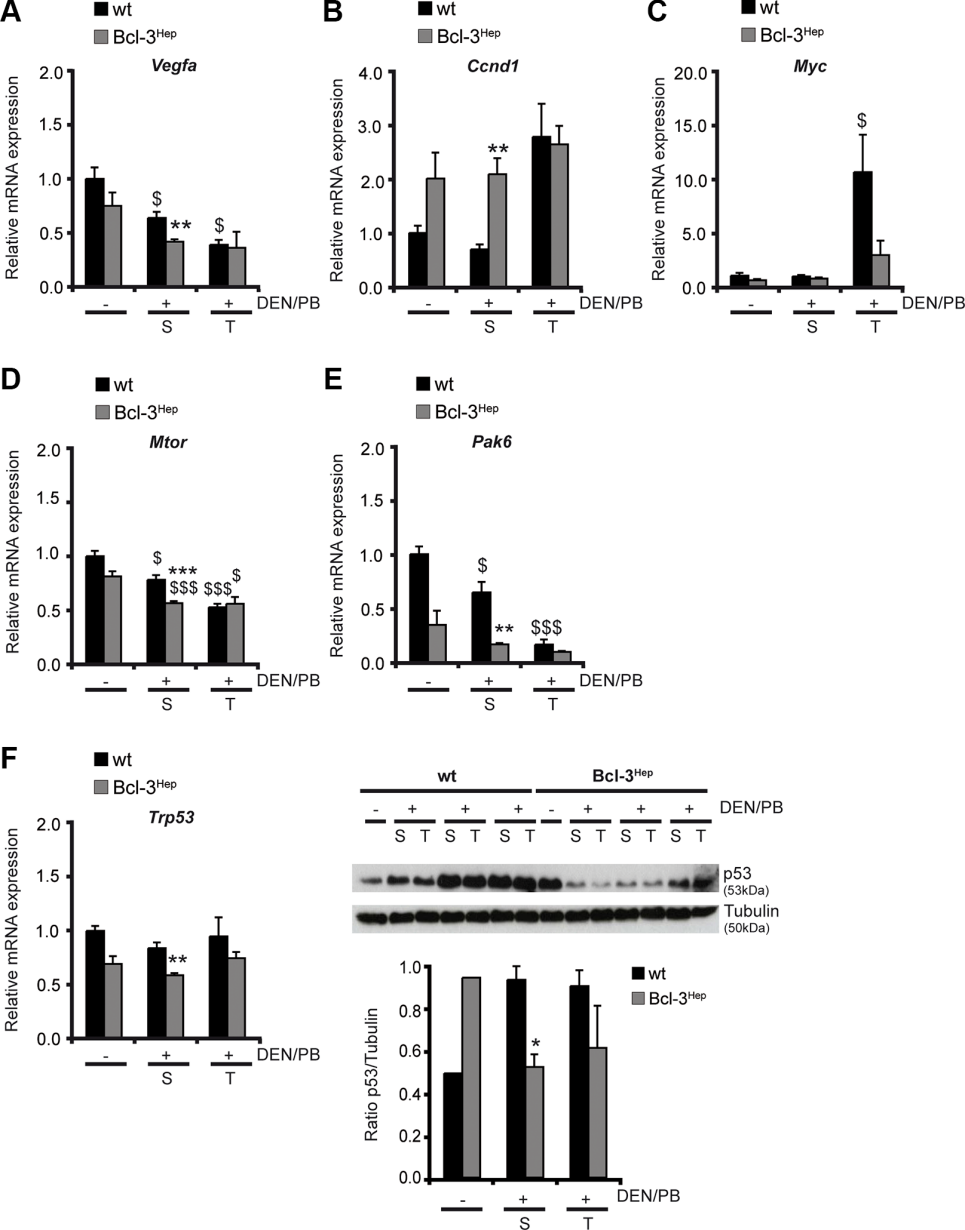
Hepatic Bcl-3 alters regulators of cell cycle control and proliferation (**A**) Relative mRNA expression of VEGF-A, (**B**) cyclin D1, (**C**) cMyc, (**D**) mTOR, (**E**) p21 and (**F**) p53 as well as immunoblotting of (F) p53 in tumor (T) and surrounding (S) tissue of DEN/PB-treated (+) mice and in the liver of untreated (−) controls. Data in A–F are means of *n* = 7–16 mice/group ± SEM. *p* values for wt vs. Bcl-3^Hep^ mice: *p < .05, **p < .01, ***p < .001 and wt/Bcl-3^Hep^ mice untreated (− DEN/PB) vs. treated (+ DEN/PB): ^$^*p* < .05, ^$$$^*p* < .001. In F representative immunoblots are shown. Tubulin served as protein loading control.

Well-defined genetic and chemical models of hepatocarcinogenesis have identified liver injury and cell death as a trigger for compensatory proliferation and hepatocarcinogenesis [6, 29]. The role of apoptosis signaling in hepatocarcinogenesis depends on the model and time point studied and increased apoptosis of HCC progenitor cells can also exert a protective effect [7]. In the DEN/PB model and in human cancer cell lines, suppression of vascular endothelial growth factor (VEGF)-A - a regulator of neovascularisation - and of the cell cycle regulator cyclin D1 resulted in protection from HCC development [30]. In DEN/PB-treated Bcl-3^Hep^ mice levels of *Vegfa* were significantly lower in the tumor surrounding tissue compared to the wild type (Figure 4A). With regards to the hepatic expression of cyclin D1, Bcl-3^Hep^ mice exhibited increased levels of *Ccnd1* in liver and tumor tissue compared to the wild type, which were unaffected by DEN/PB-treatment. In contrast, DEN/PB-induced tumor tissue derived from wild type mice exhibited a markedly increased expression of *Ccnd1* (Figure 4B).

Impaired liver cell proliferation and reduced hepatocarcinogenesis following JNK down-regulation is partially explained by reduced expression of the growth-promoter cMyc and increased expression of the growth-inhibitor CDK inhibitor p21 [30]. In agreement, Bcl-3^Hep^ mice exhibited a markedly lower expression of *Myc* in hepatic tumor tissue compared to wild type mice (Figure 4C). Also *Mtor* encoding the upstream-regulator of cMyc and cyclin D1 mammalian target of rapamycin (mTOR) was less strongly expressed in the hepatic tissue of Bcl-3^Hep^ mice compared to the wild type (Figure 4D). Following DEN/PB-treatment the hepatic expression of *Mtor* decreased to the same extent in both genotypes (Figure 4D). The expression level of *Pak6* encoding the cyclin-dependent kinase-inhibitor p21 was significantly diminished in hepatic tumor tissue of wild type mice following DEN/PB, while Bcl-3^Hep^ mice generally showed a lower expression level of p21 (Figure 4E). This could be attributed to the lower expression of its upstream-regulator p53 – encoded by *Trp53* – in Bcl-3^Hep^ mice (Figure 4F). In parallel, p53 protein was down-regulated in Bcl-3^Hep^ liver tissue in response to DEN/PB, while increased levels were detectable in the wild type (Figure 4F). These observations are in line with previous findings of suppressed p53 activation and reduced p53-induced apoptosis trough p21 from Bcl-3 [31]. A second involved signaling pathway related to cellular proliferation in HCC is Akt/PKB. Hepatic overexpression of Bcl-3 promoted Akt activity in hepatic tissue, that was almost unaffected by DEN/PB (Supplementary Figure 3A), suggesting that Akt-signaling did not influence hepatocarcinogenesis in this model. In accordance, inhibitory phosphorylation of the downstream target of Akt glycogen synthase kinase 3 (GSK3) α - also involved in control of cell proliferation and survival - was detected in Bcl-3^Hep^ livers, which was also hardly affected in response to DEN/PB (Supplementary Figure 3B). Together, these data suggest that increased Bcl-3 expression in hepatocytes inhibits cell cycle progression and proliferation in HCC and tumor-surrounding tissue suppressing proliferation and hepatocarcinogenesis.

### Hepatic Bcl-3 suppresses tumor-associated inflammation during hepatocarcinogenesis

**Figure 5:**
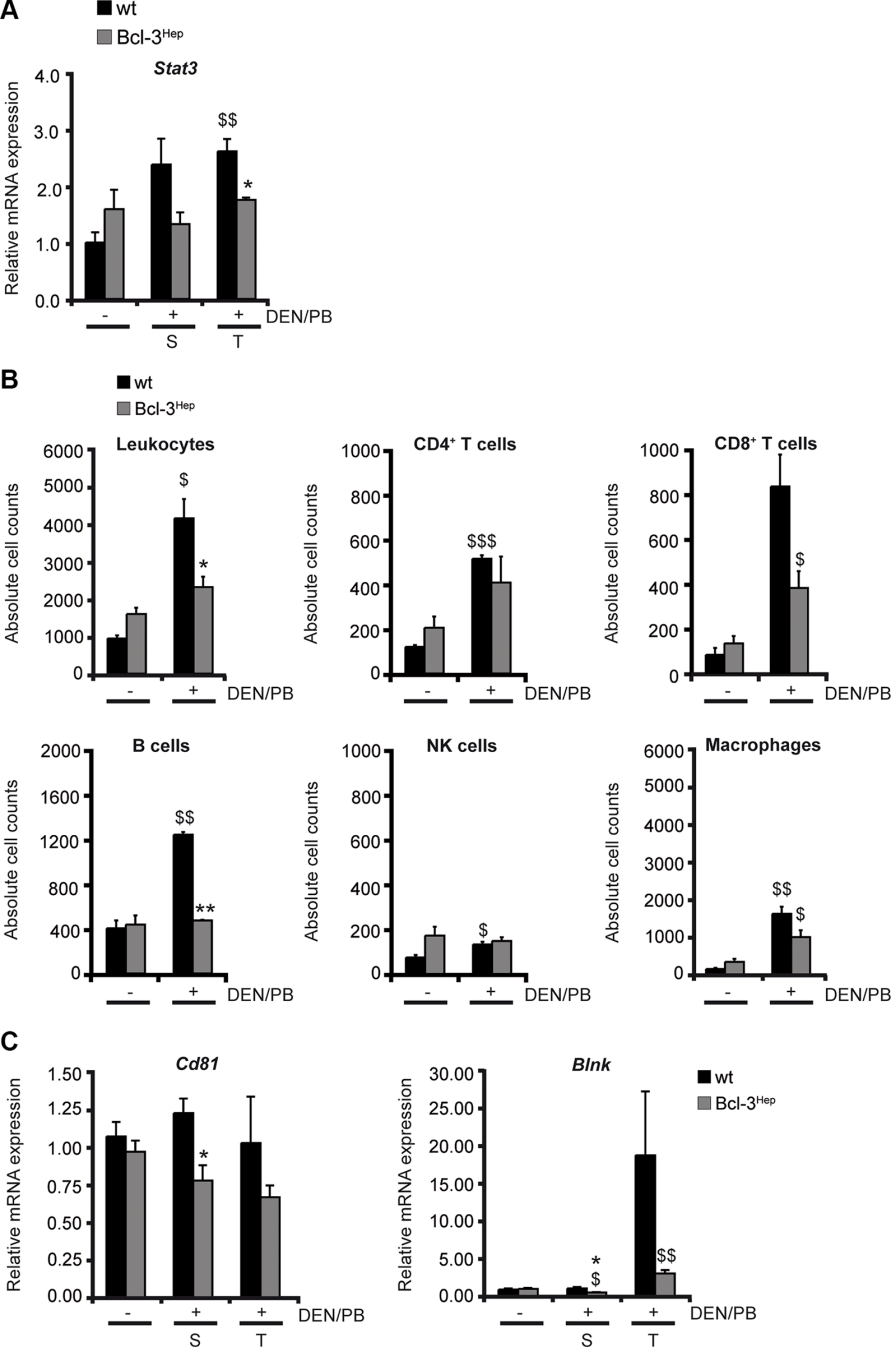
Hepatic Bcl-3 protects against DEN/PB-induced influx of immune cells to the liver (**A**) Relative mRNA expression of STAT-3 was determined by qRT-PCR in tumor (T) and surrounding (S) tissue of DEN/PB-treated (+) mice and in the liver of untreated (−) controls. (**B**) Intrahepatic immunocompetent cells derived from the liver of Bcl-3^Hep^ and wt mice were analyzed by FACS. Quantification of the different immune cell populations was performed by gating on living CD45+ leukocytes. The cell counts of CD45+CD3+CD4+ and CD45+CD3+CD8+ for T cells, CD45+CD3−CD45R/B220+ for B cells, CD45+CD3−NK1.1+ for NK cells and CD45+F4/80+CD11b+ for macrophages are depicted. (**C**) Relative hepatic mRNA expression of the B cell activation markers CD81 and BLNK. Data in A–C are means of *n* = 7–16 mice/group ± SEM. *p* values for wt vs. Bcl-3^Hep^ mice: *p < .05, **p < .01, and wt/Bcl-3^Hep^ mice untreated (− DEN/PB) vs. treated (+ DEN/PB): ^$^*p* < .05, ^$$^*p* < .01, ^$$$^*p* < .001.

HCC leads to a local inflammatory response that is triggered by cytokines/chemokines and characterized by intrahepatic accumulation of macrophages and CD8^+^ cytotoxic T cells [32]. The tumor microenvironment protects transformed cells from this immune response and on the other hand inflammation can drive neovascularization and tumor progression [33]. The signal transducer and activator of transcription-3 (STAT-3) can contribute to tumor progression by suppressing anti-tumor immunity and apoptosis, and inducing cell proliferation [34]. In wild type mice a significant elevation of *Stat3* was observed following DEN/PB that did not occur in Bcl-3^Hep^ mice (Figure 5A). Although no significant differences regarding inflammatory cytokines in the hepatic tissue or serum - including IL-1α, IL-6 and TNF – were observed between the two genotypes (data not shown), DEN/PB-induced hepatocarcinogenesis was accompanied by an increase in the absolute numbers of intrahepatic leukocytes that was augmented in wild type mice. The migrating inflammatory cells consisted primarily of macrophages, B cells and CD8^+^ T cells (Figure 5B). Previous studies have shown that the tumor microenvironment leads to the activation of B cells specific for liver-derived antigens [32]. In agreement, the mRNA expression levels of *Cd81* and *Blnk* encoding CD81 and B cell linker protein (BLNK), which are two major molecules involved in B cell activation, were markedly higher in the hepatic tissue of wild type compared to Bcl-3^Hep^ mice (Figure 5C). These data indicate an additional role of hepatic Bcl-3 in suppressing tumor-associated inflammatory processes.

## DISCUSSION

Identification of novel and potentially drugable targets for the treatment of HCC is urgently required as available treatment options are insufficiently controlling tumor progression in the majority of patients. In the current study we used a two-step model of hepatocarcinogenesis that is well established and allowed us to evaluate the role of a novel player in hepatic tissue homeostasis and hepatocarcinogenesis: Bcl-3. Bcl-3 is a transcriptional coregulator of NF-κB, which has been implicated in the pathogenesis of HCC [35]. However, the functional role of Bcl-3 in hepatocytes remains uncertain and so far only uncontrolled data is available. In patients undergoing liver resection for HCC, increased levels of NF-κB p50 homodimers and Bcl-3 were observed in the resected tumor [17], providing first evidence for its role in hepatocarcinogenesis. Also, some reports suggested that Bcl-3 overexpression provides resistance against apoptosis following DNA-damage in a transformed breast cancer cell line *ex vivo* [31].

The current study employed a well-established model using DEN as initiator and PB as promoting agent of hepatocarcinogenesis in mice. The carcinogenic capacity of DEN is related to its ability to alkylate DNA following hydroxylation by cytochrome P450 and to oxidative stress from ROS production. PB enhances the effect of DEN through induction of cytochrome P450 and causes hypermethylation of promoter regions in tumor suppressor genes influencing cellular proliferation [36]. The oncogenic potential of DEN/PB was significantly reduced in Bcl-3 overexpressing animals at 40 weeks of age. Bcl-3 reduced the number and especially the size of tumor nodules. Hepatocellular injury at this late time point was not affected by Bcl-3 expression and the predominant type of cell death was apoptosis. Interestingly, the number of dysplastic nodules and small tumors at week 40 was comparable in both genotypes. This suggests that HCC formation was reduced primarily due to effects that are occurring early following tumor initiation and that over time the number of dysplastic nodules, which are produced from continuous PB-treatment, emerge at the same rate. Therefore an earlier time point was examined and a prolonged phase of hepatocellular injury characterized by elevated ALT and hepatic expression of TNF and CCL2 was observed only in Bcl-3^Hep^ mice. It is tempting to speculate, that Bcl-3 overexpression induces cell death in oncogenic foci and transformed hepatocytes early during hepatocarcinogenesis, thus resulting in reduced tumor burden. Alternatively, a higher sensitivity towards induction of apoptosis and/or a delay in the progression from dysplastic foci to HCC could be contributing to the observed phenotype of reduced HCC. Nonetheless, the role of Bcl-3 in regulating the number of transformed, pre-neoplastic cells following DEN remains to be answered by serial analysis of several time points during hepatocarcinogenesis.

**Figure 6:**
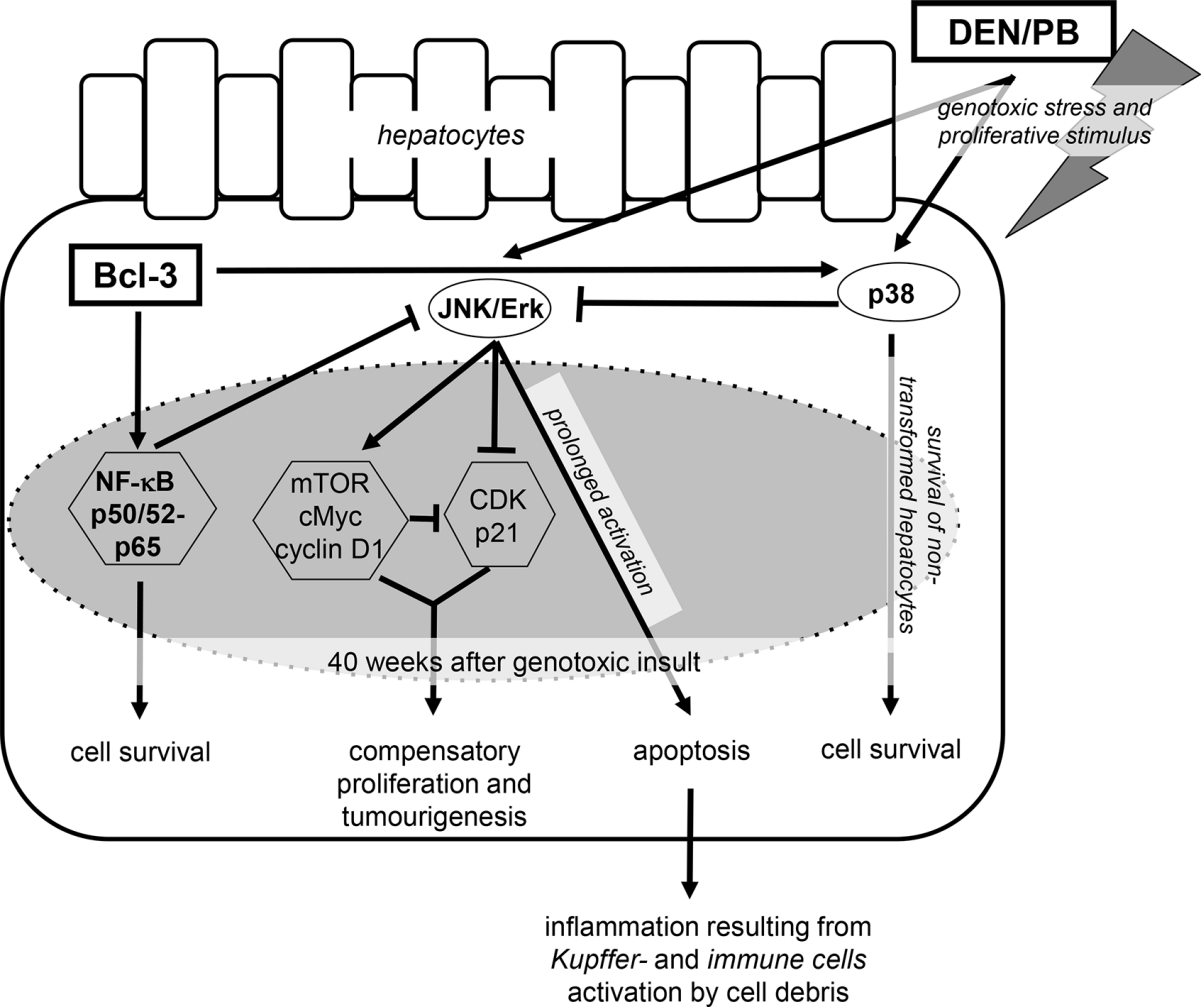
Protective effects of hepatocyte-specific Bcl-3 during DEN/PB-induced hepatocarcinogenesis Overexpression of hepatic Bcl-3 suppressed DEN/PB-induced hepatocarcinogenesis through down-modulation of the MAPK ERK and JNK and activation of the p38 and NF-κB signaling pathways, regulating hepatocellular proliferation, hepatic inflammation and cell death.

As Bcl-3 is a regulator of NF-κB signaling, p65 and MAPK signaling were examined. Interestingly, p65 and p38 were phosphorylated and activated to a higher extent in hepatic tissue and HCC in Bcl-3^Hep^ mice in response to DEN/PB. The increased NF-κB activation in Bcl-3^Hep^ mice was also reflected by elevated activity of the NF-κB subunits p50 and p52, which act as transcriptional coactivators in conjunction with Bcl-3 (see also Figure 6). As shown in several mouse and *in vitro* models, both NF-κB and p38 have cytoprotective effects and prevent prolonged JNK activation and hepatocyte death [37–39]. Mice with hepatocyte-restricted deletion of the NF-κB-coactivator IKKβ or the MAPK p38α showed excessive hepatocyte death, enhanced compensatory proliferation and augmented HCC development after DEN-exposure [31, 32]. Likewise, ablation of IKKβ enhanced activation of JNK and its down-stream transcription factor cJun leading to induction of cyclin D and cell cycle activation in hepatocytes. JNK activation is one of the driving factors in this context, as a double knock-out of IKKβ and JNK1 - but not JNK2 - prevented hepatocyte death and hepatocarcinogenesis that is driven by oxidative stress and STAT-3 activation [40, 41]. This phenotype was even more severe following deletion of IKKγ/NEMO and involved oxidative stress, prolonged JNK activation and increased cell death [6]. The Bcl-3 model exhibited diminished activation of JNK and ERK compared to the wild type at 40 weeks of age in parallel to attenuation of cell proliferation and HCC, while at the earlier time point JNK activation was enhanced in Bcl3^Hep^ mice. In the clinical context, increased JNK activation has also been observed in approximately 70% of HCCs and was associated with a poorer prognosis in patients undergoing liver resection [42]. In contrast, p38 activity is reduced in HCC and correlates inversely with tumor size, while ERK1/2 signaling is activated [43]. It was previously shown that an increased ERK/p38 ratio favors tumor growth, whereas high p38/ERK ratio induces tumor arrest (dormancy) *in vivo* and that ERK is negatively regulated by p38 [44]. Regardless of the degree of MAPK activation, liver injury at 40 weeks was only marginally different in the two genotypes.

Proliferation and cells in the in G2/M phase were significantly suppressed in Bcl-3^Hep^ mice following DEN/PB-exposure compared to the wild type. Moreover, the hepatic expression of regulators of cellular survival, proliferation and neo-angiogenesis through Bcl-3 including VEGF-A, mTOR, cyclin D1, cMyc, p53 and p21 were regulated in line with the reports on their respective function during hepatocarcinogenesis [40, 45]. A potential player in the reduction of HCC formation is cyclin D1 and it has previously been shown that Bcl-3 can activate cyclin D1 [46]. This was observed in untreated Bcl-3^Hep^ mice at 40 weeks of age, while the expression of cyclin D1 following treatment was different in wild type and Bcl-3^Hep^ mice. While DEN/PB treatment left cyclin D1 at basal levels in Bcl-3^Hep^ mice, the wild type showed a 2.8-fold induction. Moreover, cMyc - a well characterized oncogene during and a potential target for pharmaceutical interventions [36] - and its up-stream regulator mTOR were suppressed, while the CDK-inhibitor p21 - which acts to inhibit cMyc-mediated oncogenic potential [30] - was unaffected by DEN/PB-treatment in Bcl-3^Hep^ mice. Bcl-3 was shown to suppress p53 activation and to inhibit p53-induced apoptosis through p21 [31]. This was also observed in the current model, while the wild type showed significantly decreased expression of p21, increased cellular proliferation and HCC formation. Regulation of hepatocarcinogenesis in this model did not involve Akt or GSK-3 signaling. Despite increased phosphorylation of Akt and GSK-3 at baseline in Bcl-3^Hep^, no consistent changes in phosphorylation following DEN/PB between the two genotypes could be observed.

HCC is an inflammation-driven cancer and intrahepatic inflammatory cells and cytokines are involved in the pathogenesis. The release of IL-1α and cellular debris following hepatocyte death, promotes the activation of NF-κB in Kupffer cells, which in response release IL-6, IL-1β and TNF further enhancing liver cell injury and the activation of immune cells. IL-6 induces STAT-3 in neighboring hepatocytes and promotes proliferation and HCC development [33]. In the current study Bcl-3 expression in hepatocytes reduced inflammation during hepatocarcinogenesis by preventing STAT-3 expression and by decreasing the intrahepatic accumulation of macrophages and CD8^+^ T cells. Tumor-associated macrophages have been linked to cancer invasion [47], an unfavorable prognosis related to immunosuppressive actions [48], and failure to respond to the multi-tyrosine kinase inhibitor Sorafenib [49]. The role of adaptive immunity involving B cells is less clear. Some studies have shown a protective role in hepatocarcinogenesis through suppression of growth of established tumors [32], while certain B cell populations impair CD4^+^ T cell activation and thus promote tumor progression [50, 51]. Among the cytokines responsible for this effects, lymphotoxin secreted from tumor-infiltrating B cells has been implicated in tumor growth [52]. In the current study an increase in the absolute number and the activation of intrahepatic B cells was observed in the wild type. Despite the protective effects of Bcl-3 overexpression on HCC formation, no substantial difference regarding serum and liver cytokine profiles were detectable at 40 weeks.

Overall, the study adds to the role of cell death during hepatocarcinogenesis. The data supports the hypothesis that hepatocyte-specific Bcl-3 exerts protective effects by preventing DEN/PB-induced malignant proliferation and by reducing hepatic inflammation. The underlying mechanisms involve increased NF-κB p65 and p38 activation and inhibition of ERK and JNK activity at 40 weeks of age and suppression of the oncogenic potential through persistent, early cellular injury – an effect that appears to be transient and reversible. This data is encouraging and supports the exploration of Bcl-3 as a novel target in HCC therapy or its use as a prognostic marker.

## MATERIALS AND METHODS

### Animal model

All animals were bred at the animal facility of the University Medical Center Mainz, according to the criteria outlined by the “Guide for the Care and Use of Laboratory Animals” and studies were approved by the committee for experimental animal research (Landesuntersuchungsamt Rheinland-Pfalz). Bcl-3^Hep^ mice have been published [22]. Diethylnitrosamine (DEN, Sigma-Aldrich, Steinheim, Germany) dissolved in PBS was injected intraperitoneally (i.p.) in 7–10 day-old, male mice at a fix dose of 25 μg. PB was administered ad libidum dissolved in drinking water at 0.5 g/l starting at 4 weeks of age until necropsy. Controls were left untreated. Blood and liver tissue were obtained at 40 weeks of age.

### Analysis of hepatic tissue

Tumor nodules on the liver surface of all lobes were counted after sacrifice. After weighing, individual tumors, tumor surrounding tissue and normal liver tissue were separated, snap frozen and stored at −80°C for further analysis. One representative section of the left liver lobe was preserved for histological evaluation with hematoxylin and eosin (H&E) using standard protocols. Histological sections were evaluated blinded by an experienced hepato-onco histopathologist (TL). Pictures were taken using an Olympus BX51 microscope and the Olympus Image Analysis Software anySIS docu.

### Serological analysis

Serum was obtained by cardiac puncture from anesthetized mice and serum alanine-aminotransferase (ALT), aspartate aminotransferase (AST) and lactate dehydrogenase (LDH) levels were measured using a standard clinical analyzer (Hitachi 917, Roche, Mannheim, Germany).

### Immunoblotting and immunohistochemistry

Primary antibodies included: Akt, p-Akt, Bcl-xL, ERK, p-ERK, GSK-3α/β, p-GSK-3α/β, JNK, p-JNK, NF-κB p65, p-NF-κB p65, p38 and p-p38 (all Cell Signaling Technology Inc., Danvers, MA, USA), p53 (Santa Cruz Biotechnology, Santa Cruz, CA, USA) and α-tubulin (Sigma-Aldrich). Membranes were exposed to anti-mouse, anti-goat (both DAKO Denmark A/S, Glostrup, Denmark) or anti-rabbit (Santa Cruz Biotechnology) secondary antibodies conjugated with horseradish peroxidase. Densitometric analyses were performed using ImageJ software.

Immunohistochemistry for activated (cleaved) caspase 3 (Cell Signaling Technology Inc.) was performed as previously described [53].

For immunohistochemical staining of Ki-67 deparaffinized sections were hydrated in a graded series of alcohol solutions. Sections were incubated for antigen retrieval (boiling the sections at 95°C for 15 minutes in 10 mM sodium citrate buffer (pH 6.0)) and treated with 3% H_2_O_2_ to block endogenous peroxidase. Monoclonal rat anti-mouse Ki-67 antibody (eBioscience) was applied on the slides and incubated overnight in a humid chamber in refrigerator at 4°C. The secondary rabbit anti-rat biotinylated antibody was applied (eBioscience) for 15 minutes at room temperature on the next day, followed by incubation with streptavidin peroxidase (DAKO Corp.). Sections were washed with TBS/0.1% Triton three times after each step. Staining was performed with diaminobenzidine chromogen solution (DAB, DAKO Corp.), counterstained with hematoxylin. Ki-67 positive cells and counted in a blinded manner in at least 40 random visual fields (magnification: 100×).

### Isolation of primary hepatocytes by collagen perfusion and *ex vivo* stimulation

Hepatocytes were isolated as previously described [54]. For stimulation hepatocytes were plated in collagen-coated culture plates (5 × 10^5^/1.5 ml) in DMEM supplemented with 10% FCS, 2 mM L-glutamine, 100 U/ml penicillin/streptomycin, 1 mM MEM sodium pyruvate, 25 mM D-glucose, 20 mM Hepes (all from Gibco, Grand Island, NY, USA), 40 IE/ml insulin (Lilly, GieΔen, Germany) and 10^−6^ M lithocolic acid (Carl Roth, Karlsruhe, Germany). After 24 hours medium was changed and hepatocytes were treated with DEN (Sigma-Aldrich) for further 24 hours. If indicated, 50 μM zVAD, 100 μM SP600125 (both from Enzo Life Sciences, Lörrach, Germany), 10 μM SB203580 (Sigma-Aldrich) or 10 μM BAY 11-7082 (Calbiochem, EMD Chemicals, Inc. San Diego, CA, USA) was added one hour before DEN-treatment. Cell survival was assessed by MTT assay (Sigma-Aldrich).

### Quantitative real-time PCR

Isolation of total RNA, cDNA synthesis and qRT-PCR were performed as previously described [53]. Roche LightCycler software (LightCycler 480 Software Release 1.5.0) was used to perform advanced analysis relative quantification using the 2^(−ΔΔC(T))^ method. Expression data were normalized to the housekeeping gene GAPDH and the mean of untreated wild type mice was considered 1. Primer sequences for *Bcl2*: forward: GCCAGGGAAGATGGCTGAGTCTG, reverse: TTGGA GCCGACTCAAAGGCGGG, *Bcl2l1*: forward: GGGGTC GCATCGTGGCCTTT, reverse: AAGCGCTCCTGGCCT TTCCG, *Blnk*: forward: ATGGACAAGCTGAA, reverse: TTATGAAACCTTCA, *Ccl2*: forward: CTT CTGGGCC TGCTGTTCA, reverse: CCAGCC TACTCATTGGGAT CA, *Cd81*: forward: CGCCAAGGATGTGAAGCAGTTC, reverse: TCCCAGAGAAGAGCTCATCGAT, *Myc*: forward: AACGAAAAGGCCCCCAAGGTAGTGATCC, reverse: GTCGTTTCCTCAATAAGTCCTTTTC, *Ccnd1*: forward: TGCCATCCATGCGGAAA, reverse: AGCGG GAAGAACTCCTCTTC, *Mki67*: forward: TCTGATGTT AGGTGTTTGAG, reverse: CACTTTTCTGGTAACTTCT TG, *Mtor*: forward: CTGGGGCTCAAGTGTGTGCAGT TC, reverse: GAACTATTGGGTGAATGATGCGGG, *Pak6*: forward: GGCCCGGAACATCTCAGG, reverse: AA ATCTGTCAGGCTGGTCTGC, *Stat3*: forward: GGAGGA GCTGCAGCAGAAAG, reverse: TGTGTTCGTGCCC AGAATGT, *Tnf*: forward: GAA GTT CCC AAA TGG CCT CC, reverse: GTG AGG GTC TGG GCC ATA GA, *Trp53*: forward: CACGTACTCTCCTCCCCTCAAT, reverse: AA CTGCACAGGGCACGTCTT, *Vegfa*: forward: CTGTGC AGGCTGCTGTAACG, reverse: GCTCATCTCTCCTATG TGCTGGC, *Xiap*: forward: CGACGCTAATCGAGGGC CGC, reverse: TCGCGCCAAGCACTCCAGTC.

### Determination of oxidative stress

Malondialdehyde levels were determined from whole liver tissue according to the manufacturer's instructions (Lipid Peroxidation (MDA) Assay Kit, BioVision, Mountain View, CA, USA).

### Determination of caspase 3 activity

Caspase 3 activity was determined following lysis of liver tissue (lysis buffer: 20 mM Tris/HCl pH 8.0, 5 mM EDTA, 0.5% Triton X, cOmplete Mini protease inhibitor cocktail (Roche, Indianapolis, IN, USA)). 50 μl of tissue lysate (1.2 μg/ml) were added to a reaction mixture containing 50 μl 2× reaction buffer (50 mM HEPES pH 7.5, 100 mM NaCl, 20% glycerol, 0.1% 3-[(cholamidopropyl-)dimethylammonio]-1-propanesulfonate (CHAPS), 10 mM DTT) and 5 μl 1 mM fluorogenic peptide substrate acetyl-DEVD-AFC (AC-DEVD-AFC) (Biomol, Hamburg, Germany) and incubated at 37°C for 1–2 hours without light. Production of AFC was measured in a spectrofluorometer with an excitation wavelength of 405 nm and an emission wavelength of 535 nm.

### Determination of the NF-*κ*B activity

NF-*κ*B activity was measured using the TransAM NF-*κ*B Family Kit according to the manufacturer's instructions (Active Motif, Carlsbad, CA, USA).

### FACS analysis of intrahepatic leukocytes

Intrahepatic leukocytes were isolated and subjected to flow cytometric analysis (FACS) as previously described [54].

### Statistical analysis

Values are given as mean ± standard error of the mean (s.e.m.) of *n* = 14 Bcl-3^Hep^ mice, *n* = 16 wild type littermates and *n* = 7 untreated control mice per genotype. The *F*-test was used to verify the assumption of equal variances, and two-tailed Student's *t*-test was used to determine statistical significance. Statistically significant values are presented as: *^/$^*p < .05*, **^/$$^*p < .01*, ***^/$$$^*p < .001*.

## SUPPLEMENTARY MATERIALS FIGURES


