# Supplementary material for: Hepatic B cell leukemia-3 suppresses chemically-induced hepatocarcinogenesis in mice through altered MAPK and NF-κB activation
Source: Oncotarget. 2016 Jul 28;8(34):56095–109. doi: 10.18632/oncotarget.10893 (PMC5593547; doi:10.18632/oncotarget.10893)
Supplement: Supplementary file 1 [file oncotarget-08-56095-s001.pdf]

Hepatic B cell leukemia-3 suppresses chemically-induced hepatocarcinogenesis in mice through altered MAPK and NF-κB activation

Supplementary Materials

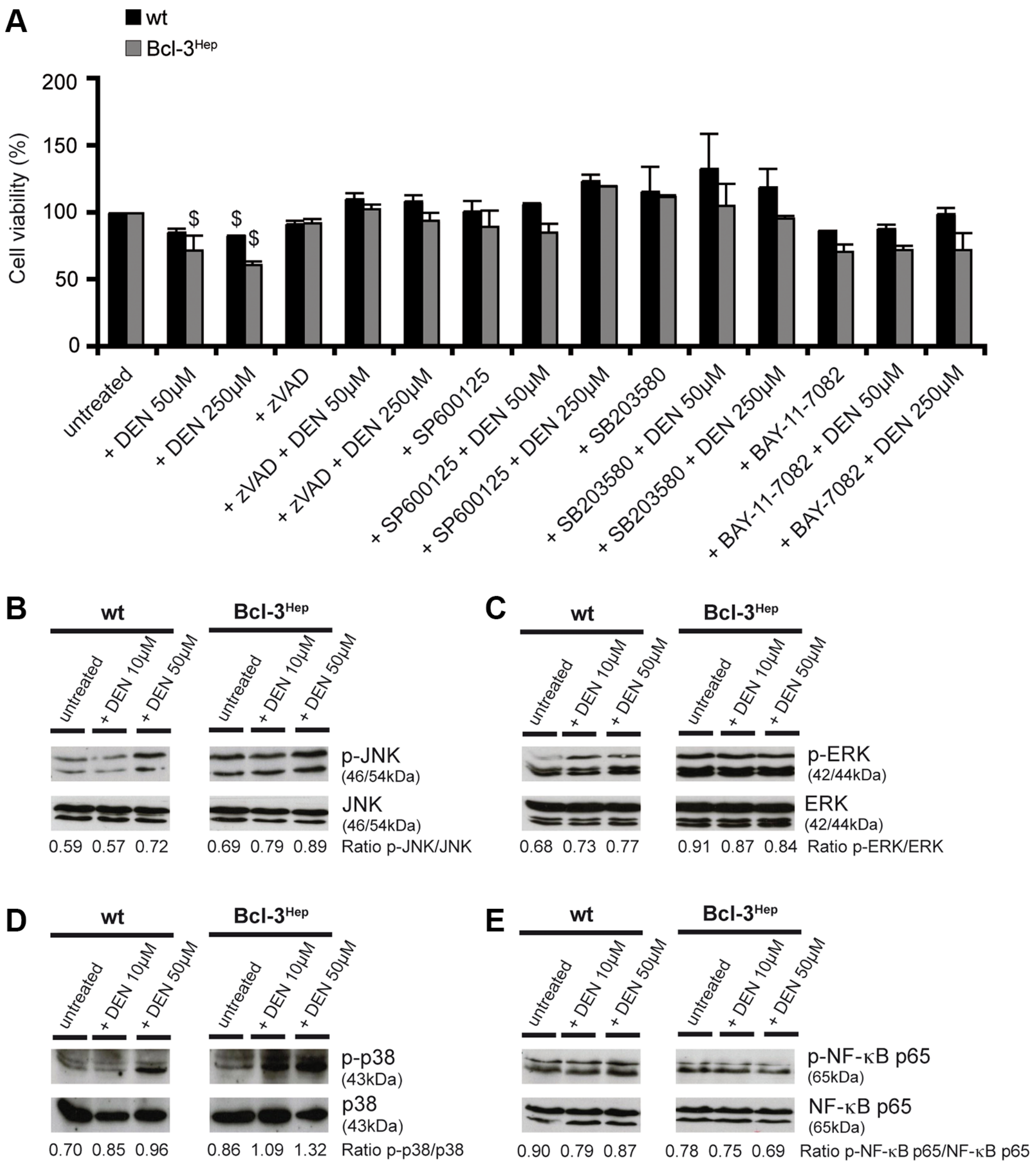

Supplementary Figure S1: *Ex vivo* treatment of with DEN promotes apoptosis and MAPK activation in Bcl-3^{Hep} hepatocytes. Hepatocytes isolated by collagen perfusion of whole liver tissue of Bcl-3^{Hep} and wt mice were treated *ex vivo* with DEN as indicated and if indicated with 50 µM zVAD, 100 µM SP600125, 10 µM SB203580 or 10 µM BAY 11-7082 for 24 h. (**A**) MTT was used to measure relative cell viability compared to untreated samples. Activation of (**B**) JNK, (**C**) ERK, (**D**) p38 and (**E**) NF-κB p65 was determined by immunoblotting of phosphorylated and total p38/JNK/ERK/NF-κB p65 protein. In A mean + SEM of duplicates from 3 independently performed experiments are shown. *p* values for Bcl-3^{Hep}/wt hepatocytes untreated (−) vs. treated (+DEN): $^{\$}p < .05$. In B–E representative immunoblots are shown.

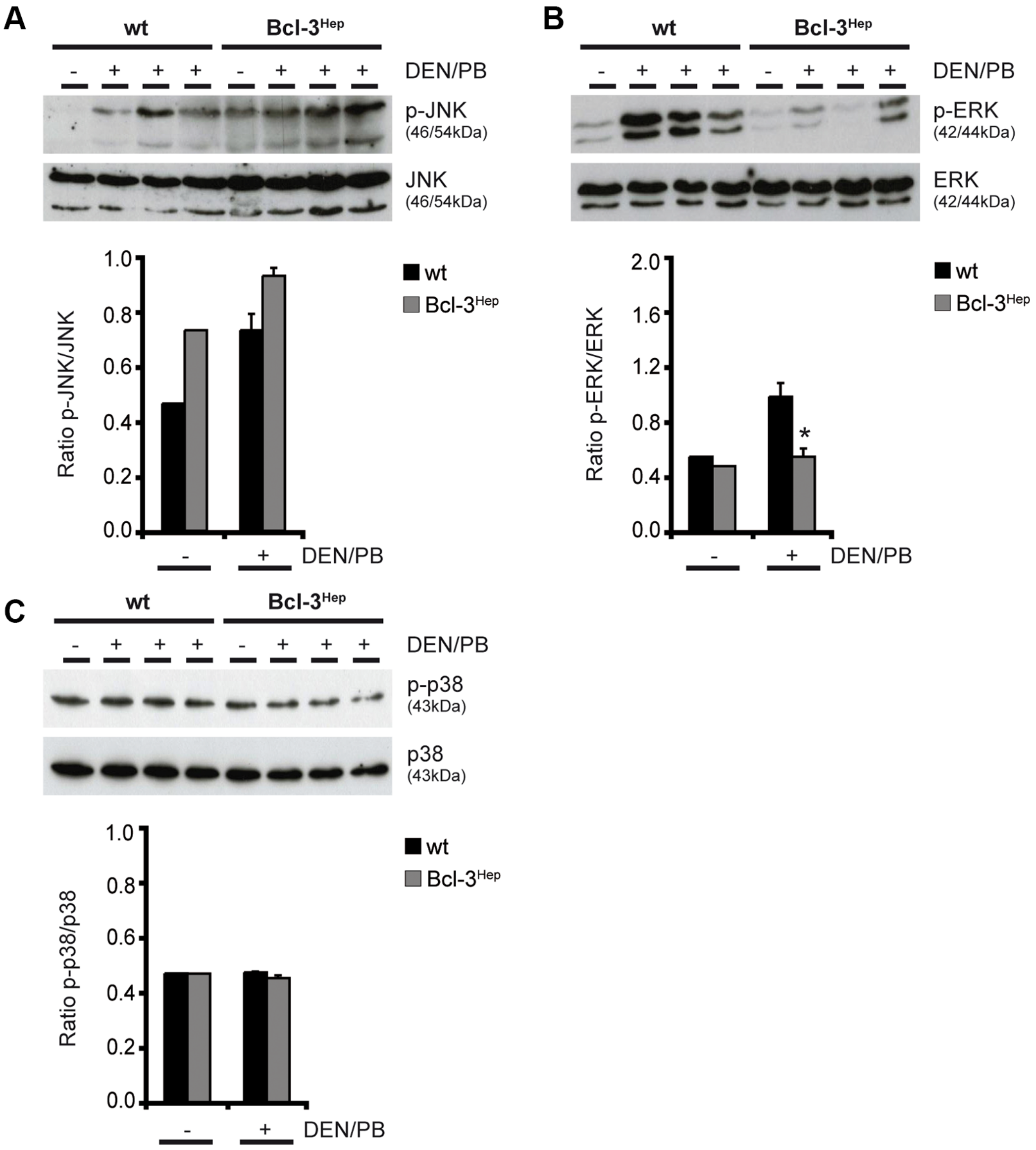

Supplementary Figure S2: Altered MAPK-signaling in DEN/PB-treated Bcl-3^{Hep} mice at 7 weeks of age. (**A**) Activation of JNK, (**B**) ERK and (**C**) p38 and was determined by immunoblotting of phosphorylated JNK/ERK/p38 and total JNK/ ERK/p38 protein in liver tissue of 7-weeks-old DEN/PB-treated Bcl-3^{Hep} and wt mice and in the liver of untreated controls. In A–C representative immunoblots are shown. *p* values for wt vs. Bcl-3^{Hep} mice: $*p < .05$.

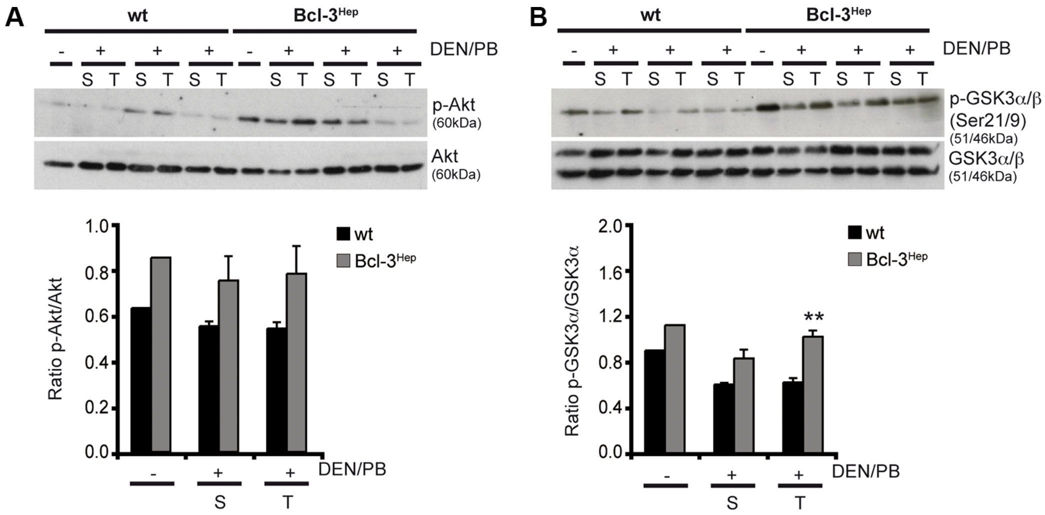

Supplementary Figure S3: Alterations of Akt and GSK3 phosphorylation in Bcl-3^{Hep} mice at 40 weeks of age following DEN/PB-exposure. (**A**) Activation of Akt and (**B**) GSK-3α/β was determined by immunoblotting of phosphorylated Akt/ GSK-3α/β and total Akt/ GSK-3α/β protein in tumor (T) and surrounding (S) liver tissue of 40-weeks-old DEN/PB-treated Bcl-3^{Hep} and wt mice and in the liver of untreated controls. In A and B representative immunoblots are shown. *p* values for wt vs. Bcl-3^{Hep} mice: $**p < .01$.
